# Spread of virus laden aerosols inside a moving sports utility vehicle with open windows: A numerical study

**DOI:** 10.1063/5.0061753

**Published:** 2021-09-15

**Authors:** Nirvik Sen, K. K. Singh

**Affiliations:** 1 Chemical Engineering Division, Bhabha Atomic Research Centre, Trombay, Mumbai 400085, India; 2 Homi Bhabha National Institute, Anushaktinagar, Mumbai 400094, India

## Abstract

A three dimensional Computational Fluid Dynamics (CFD) model to study the dispersion of virus laden aerosols in a car moving with its windows open is reported. The aerosols are generated when a possibly infected passenger speaks. A sports utility vehicle having three rows of seats has been considered. As the vehicle moves forward, its interior will exchange air from the surroundings. The CFD model captures the flow patterns generated both outside and inside the vehicle. This internal aerodynamics will in turn dictate how aerosols will spread across the interior and whether or not they will be transported outside the vehicle. A Lagrangian approach is used to determine the transport of the aerosol particles and the effect of particle size on the simulation result has been studied. Four sets of scenarios of practical interest have been considered. The first set shows the effect of vehicle speed on aerosol transport, and the second set describes what happens when some of the windows are closed, while the third set describes how aerosol transport is affected by the location of the passenger speaking. The fourth set describes how a gush of cross wind affects aerosol transport. Simulation results reveal that when all windows are open, aerosols can go out of one window and then return back to the vehicle interior through another window. Results also reveal that when a passenger sitting in the second row speaks, the aerosols generated span across the entire volume of the car interior before going out through the open windows.

## I. INTRODUCTION

Even after almost one and a half years, COVID-19 rages on. Although some countries have been able to turn the tide thanks to vaccination, the pandemic rages on in many parts of the world. This has made COVID-19 arguably one of the worst crises in the history of mankind in the recent past. As of today (24 June 2021), the world has witnessed more than 180 million cases of COVID-19 and more 3.9 million people have lost their lives (Worldometer, 2021).

Since last year, COVID-19 has led to widespread research efforts not only in medical science, economics, and virology, but also in many other fields, for example pedagogical science (Tiejun, 2021), meteorology (Kerr *et al.*, 2021), psychology and sociology (Dubey *et al.*, 2020), environmental science (Fattorini and Regoli, 2020), and sustainable development (Megahed and Ghoneim, 2020). One such field is fluid mechanics. The virus may be transmitted from one person to another by means of respiratory droplets and/or aerosols. Well established numerical techniques like computational fluid dynamics (CFD) can be used to determine how such respiratory drops and/or aerosols spread in different settings and situations based on the prevalent aerodynamics (Mittal *et al.*, 2020; Peng *et al.*, 2020). Thus, numerically, it is possible to track the movement of very small (micron-size) drops/particles which are released when a person sneezes, coughs, speaks, or even breaths (Pendar *et al.*, 2020; Liu *et al.*, 2021; Agrawal and Bhardwaj, 2021; Agrawal and Bhardwaj, 2020; Wang *et al.*, 2020). Today, aerosol-based transmission is considered as a possible manner of spread of COVID-19 virus (Greenhalgh *et al.*, 2021). Unlike large respiratory droplets which tend to quickly fall to the ground, small aerosol particles can remain suspended in the air for a long duration of time, thereby increasing the chances of infection. Moreover, in the presence of air currents, such small particles can be transported over a large distance. As the span and strength of these air currents will be different for different scenarios/settings, CFD has become a very powerful tool to predict the air flow patterns for a specific setting (Burgmann and Janoske, 2021; Abuhegazy *et al.*, 2020) and thus provides an estimate of aerosol transmission in that setting, thereby quantifying the associated risk of transmission. Experimental and numerical techniques have also been reported, which quantifies the effectiveness of mitigation techniques, like using face masks, face shields, etc. (Verma *et al.*, 2020; Arumuru *et al.*, 2020; Prasanna Simha and Mohan Rao, 2020; Agrawal and Bhardwaj, 2020).

Modeling of the movement and dispersion of micrometer-size aerosols for different scenarios has been reported extensively in the literature, especially in the wake of the present pandemic. Significant research has been carried out to develop numerical models that focus on how cough induced respiratory droplets spread in a given setting (Dbouk and Drikakis, 2020a; Nazari *et al.*, 2021; Wu *et al.*, 2021; Sen, 2021; Sen and Singh, 2021). There have also been quite a few studies that focus on how aerosols (which is what is left after the volatile component of respiratory droplets have evaporated) spread in a variety of settings. Abuhegazy and co-workers reported a CFD model to study dispersion of aerosols inside a classroom (Abuhegazy *et al.*, 2020). The authors studied the effect of opening windows and quantified how air purifiers help in reducing the chances of transmission. Liu and co-workers simulated how aerosol (virus laden) particles are dispersed inside an air conditioned restaurant (Liu *et al.*, 2021). The authors used Large Eddy Simulation (LES) method to track the turbulence induced inside a restaurant. Narayanan and Yang reported a CFD based model that can predict dispersion and transmission of aerosols inside a music class (Narayanan and Yang, 2021). The authors studied the effect of different aerosol generation rates and the effect of location of air purifiers. Dbouk and Drikakis (Dbouk and Drikakis, 2021) reported a Euler–Lagrangian CFD model to study the airborne transmission of virus in an elevator. The authors studied the effect of position and operation of the inlets and outlets of the air ventilation system inside the elevator. They also studied the effect of installation of an air purifier inside the elevator, and their results showed that even though an air purifier somewhat reduces the transmission, it does not eliminate the problem.

Quite a few studies on spread and transmission of droplets and/or aerosols inside different means of public transport have been reported. Such studies are vital considering the fact that with the opening of the economy, more and more people will start using public transport. Yang and co-workers used CFD models to study the transmission and evaporation of micrometer-size droplets inside a coach bus (Yang *et al.*, 2020). Zhang and co-workers simulated the spread of aerosols inside a bus (Zhang *et al.*, 2021). Zhang and Li developed a numerical model to study the dispersion of cough droplets inside a high-speed rail cabin (Zhang and Li, 2012). Elmaghraby and co-workers reported a numerical model to study the spread and dispersion of airborne contaminants inside a passenger aircraft cabin while the aircraft ascends (Elmaghraby *et al.*, 2020). Talaat and co-workers simulated the dispersion and transmission of aerosols inside a Boeing 737 aircraft (Talaat *et al.*, 2021). Recently, Mathai and co-worker reported a CFD model with a species transport equation to model the spread of pathogenic species inside a car (Mathai *et al.*, 2021). They modeled two persons seated diagonally opposite inside the car. They studied the effect of opening the windows on transmission of the pathogenic species.

The cited studies in the previous paragraph show that governing equations used to model the prevailing aerodynamics for a given setting as well as those to model the transport of aerosol particles are fairly well known. In this work, we report the results of the numerical solution of these equations in yet another setting of practical interest, i.e., inside a moving sports utility vehicle (SUV) with its windows open. In a majority of the works reported on the dispersion of virus laden aerosols inside public transport, the setting is typically a closed space be it a high speed rail, a bus, or an airplane. However, when a car is moving with its windows open, there will be a significant exchange of air. Air from outside will come inside the vehicle, while that inside the vehicle will go out. In spite of the vast quantity of work that has been reported so far in aerosol transmission, such cases where the interior of a vehicle interacts with the surrounding are rare. The only departure to this is the work by Mathai *et al.* (2021), where they did consider the effect of air exchange with the surrounding. However, the authors used a species transport equation to model the spread of pathogenic species and not a Lagrangian approach. In this work this gap has been addressed.

Aerosols can be generated while coughing, speaking, and breathing. The respiratory droplets will undergo evaporation. The rate of evaporation depends on the difference in water vapor concentration in the saturated film surrounding the droplet and that in the bulk air which in turn will depend on the ambient temperature and relative humidity. Recently, a novel unsteady state theory of evaporation which considers a thermal history kernel and provides transient Nusselt (Nu) and Sherwood (Sh) numbers as a function of the Reynolds (Re), Prandtl (Pr), and Schmidt numbers (Sc), has been reported (Dbouk and Drikakis, 2020c). The authors implemented this new theory in a CFD based model to understand the effect of weather conditions on the transmission of virus laden droplets. Eventually, the entire volatile content of the droplet will be lost, and only a particulate aerosol comprising the crystallites of the insoluble components of respiratory droplets will remain. However, it has been reported that the evaporation step is quicker than the time span for which aerosol particles can remain suspended in an air stream (Liu *et al.*, 2017; Narayanan and Yang, 2021; Morawska, 2006). In this present work, we have considered the dispersion and transmission of aerosols which are generated while a person speaks. As timescale of evaporation is very small, it has been neglected, and transport of aerosols has only been considered. Breakage and/or coalescence of aerosol particles is also not considered. Hence, the size/diameter of the particles does not change with time and space. A similar approach has been used in many studies (Abuhegazy *et al.*, 2020; Narayanan and Yang, 2021). At this stage, it is important to point out that as we are not modeling the phenomenon of evaporation of released saliva droplets, we will be losing some information on the state of dispersion of the droplets (which will be of bigger size compared to aerosols) closer to the person who is speaking.

In this study, we analyze the dispersion and transmission of aerosols inside a SUV moving with its windows open. Particular focus of the study is to learn how aerosol dispersion is affected by the exchange of air within the vehicle and outside, which in turn is effected by vehicle speed and degree of opening of the windows. Furthermore, the effect of source of aerosol generation and effect of cross winds on the nature of aerosol dispersion has also been presented. The numerical model used for the analysis is premised on some assumptions. It is assumed that the aerosols are generated due to an unhindered and continuous speaking person not wearing a mask. Ejection of aerosols is in a horizontal plane, and the diameter of the aerosol is fixed. These assumptions have been commonly used in similar studies, albeit in different settings (Abuhegazy *et al.*, 2020; Narayanan and Yang, 2021).

## II. NUMERICAL MODEL

### A. Computational domain

**FIG. 1.**
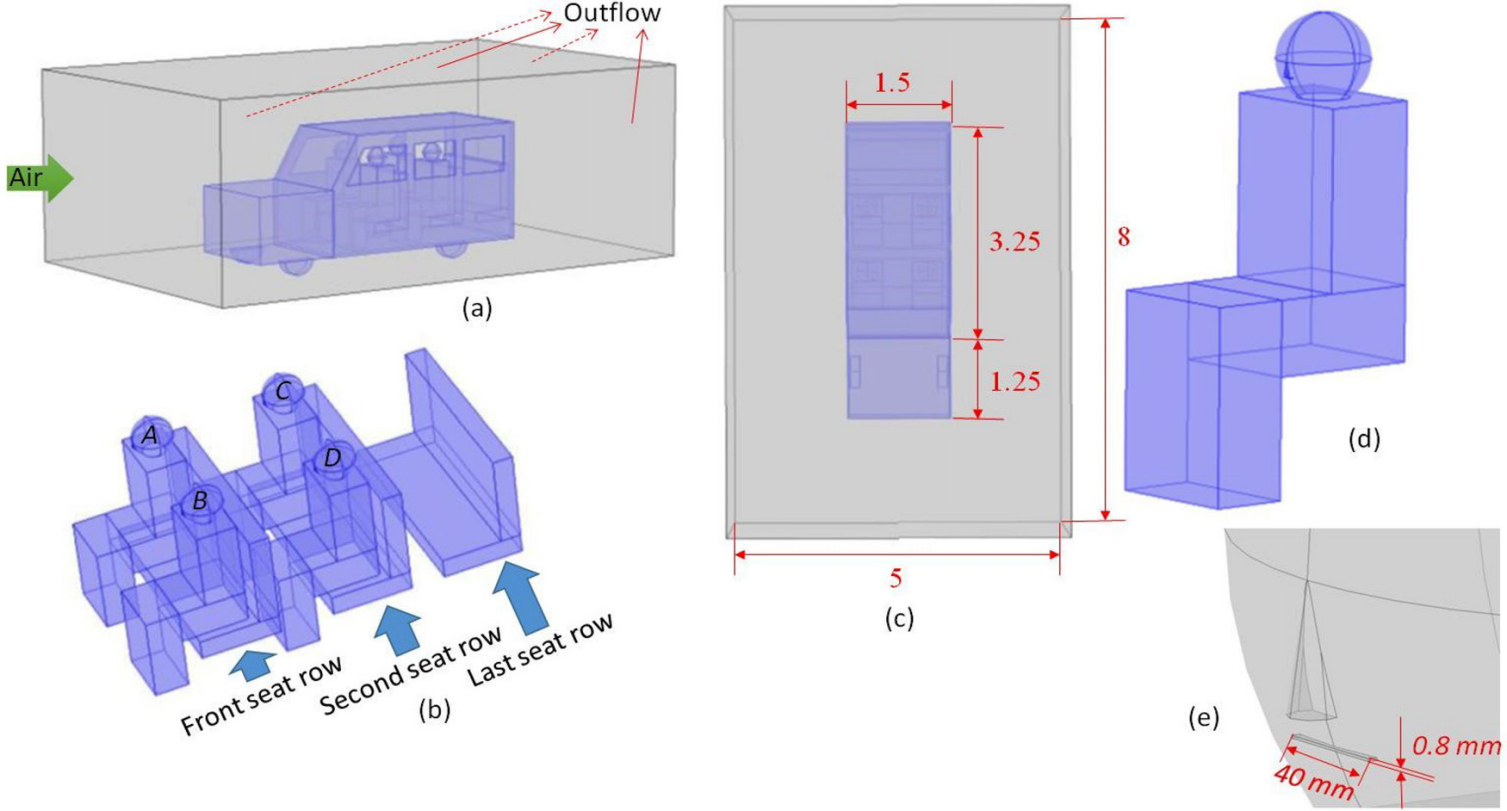
A typical computational domain used in the numerical simulations. (a) shows the isometric view of the overall domain, (b) shows the isometric view of the seating arrangement along with passengers, (c) shows the top view along with relevant dimensions, (d) shows one seated passenger, and (e) shows close-up of the face of a passenger.

Figure 1 shows the computational domain used in the preset work is shown. A six seater SUV with three rows of seat has been considered. The windows of the SUV are open, which ensures air inside and that outside the vehicles are exchanged. Thus, the computational domain comprises of both the vehicle and a portion of the surrounding. The surrounding enclosure is a rectangular box (L × B × H = 8 × 5 × 3 m^3^) having dimensions greater than the vehicle (L × B × H = 4.5 × 1.5 × 1.5 m^3^). Inside the car, four persons are seated, two in each row. The third row is empty. The dimensions of the car interior and the person seated inside are typical as applicable in real life setting. For the sake of simplicity, the roof and the back of the SUV are considered flat. The angle of inclination of the windscreen in the front is 30° with vertical. Simulations with different degrees of opening of window are carried out. A simplistic representation of human body (in seated posture) has been used to keep the computational time within reasonable limits. A rectangular mouth print has been considered (Dbouk and Drikakis, 2020a; Dbouk and Drikakis, 2020b).

### B. Physics in the numerical model

An Euler–Lagrangian technique has been used in this work. The flow field of air prevalent inside and outside the SUV is first modeled in an Eulerian reference frame. As the vehicle moves at a reasonably high speed and because of the boundary layer separation behind the vehicle significant turbulence in air is expected, there will also be significant boundary layer separation, which will lead to recirculations and thereby a significant degree of turbulence inside the vehicle. Turbulence in the computational domain has been captured using standard k–ε model. Such a turbulence treatment has been widely used for scientific, industrial, and automotive applications (Mathai *et al.*, 2021; Bardina *et al.*, 1997). Table I below lists the governing equations used in this work. The micrometer-size aerosols injected into the computational domain due to a person speaking are treated as discrete particles using a Lagrangian approach. The spatial movement of aerosol particles due to prevailing aerodynamics is captured by integrating the equation of motion of each particle. Gravitational and drag forces acting on the particles have been considered. Drag force has been quantified using Schiller–Naumann drag law (Sen 2021; Sen and Singh, 2021).

**TABLE I.** The governing equations solved.

$\nabla .\left(\vec{u}\right)=0,$	Continuity equation	(1)
$\rho \frac{\partial \vec{u}}{\partial t}+\rho \left(\vec{u}.\nabla \right)\vec{u}=\nabla .\left[-pI+\overset{̿}{\tau }\right]+\rho_{c}\vec{g},$	Momentum equation	(2)
$\overset{̿}{\tau }=\left(\mu +\mu_{T}\right)\left(\nabla \vec{u}+{\left(\nabla \vec{u}\right)}^{T}\right),$	Closure equation for momentum equation	(3)
$\frac{\partial }{\partial t}(\rho k)+\nabla .(\rho \vec{u}k)=\nabla .\left(\frac{\mu_{t}}{\sigma_{k}}\nabla k\right)+G_{k}-\rho \varepsilon ,$	Conservation of turbulent kinetic energy (k)	(4)
$\frac{\partial }{\partial t}(\rho \varepsilon )+\nabla .(\rho \vec{u}\varepsilon )=\nabla .\left(\frac{\mu_{t}}{\sigma_{\varepsilon }}\nabla \varepsilon \right)+\frac{\varepsilon }{k}\left(C_{1\varepsilon }G_{k}-C_{2\varepsilon }\rho \varepsilon \right),$	Conservation of turbulent energy dissipation rate (ε)	(5)
$\mu_{t}=\rho C_{\mu }\frac{k^{2}}{\varepsilon }C_{\mu }=0.09,$	Closure to Eqs. (4) and (5): turbulent viscosity term.	(6)
$G_{k}=\mu_{t}\left(\nabla \vec{u}+\nabla {\vec{u}}^{T}\right)\;\;:\;\;\nabla \vec{u},$	Closure to Eqs. (4) and (5): generation of turbulent kinetics energy.	(7)
$m_{d}\frac{d\vec{u_{d}}}{dt}=\left(\rho_{d}-\rho \right)g+\frac{C_{D}\pi d_{d}^{2}\rho }{8}\left|\vec{u_{d}}\left.-\vec{u}\right|\left(\vec{u_{d}}-\vec{u}\right)\right.,$	Equation of motion of drop	(8)
$C_{d}=\text{max}\left\{\frac{24}{{Re}_{d}}\left(1+0.15{Re}_{d}^{0.687}\right);0.44\right\},$	Closure to Eq. (8); drag law	(9)

### C. Initial, boundary conditions, and numerical approach

The flow of air generated inside the vehicle is purely due to the opening of the windows and influx of the surrounding air. The windows essentially represent the opening through which the vehicle interior and surrounding enclosure are connected. The vehicle has been assumed to be at rest, while an air flow equal to the vehicle velocity has been assigned to the front surface of the enclosure. Thus, the front surface is defined as velocity inlet. The remaining surfaces (except the bottom most surface) have been defined as outflow. The four persons are seated in the first two rows of seat and are defined as A, B, C, and D as shown in Fig. 1. All other surfaces of the vehicle and persons seated there are defined as wall.

The mouth of a person has been modeled as a rectangular slit (Dbouk and Drikakis, 2020a). In reality, there may be distribution of particle sizes of the aerosol as the parent respiratory drops have a drop size distribution. However, a majority of reports suggest that aerosol particles of interest are typically in the range of 1–10 *μ*m (Narayanan and Yang, 2021). We have considered a fixed particle size of the aerosol in this work in line with the approach used in many similar works (Narayanan and Yang, 2021; Abuhegazy *et al.*, 2020). However, we have also studied how particle diameter (1.5–150 *μ*m) affects the movement and state of dispersion of the particles. The aerosols ejected during speaking are quite dilute (∼500–2000 particles per liter of ejected airflow) based on experimental observations (Shao *et al.*, 2021). Because of this, it may be assumed that the presence of particles will not alter the flow field, and hence only one way coupling between the Lagrangian particles and the flow field established in the Eulerian frame of reference is considered (Abuhegazy *et al.*, 2020).

Steady state simulations are first carried out to determine the flow field generated as the vehicle moves forward at a given speed and with a given degree of window opening. As we have not modeled evaporation, temperature and species balance equations (for water vapor) have not been solved. Once steady state velocity and turbulence fields are generated, aerosol particles are injected into the domain from the mouth of a given person. The number density of particles coming out of the mouth while speaking is considered to be 570 particles per second (Narayanan and Yang, 2021; Alsved *et al.*, 2020). The velocity with which these particles are ejected is considered to be 0.16 m/s (Narayanan and Yang, 2021). After being released, these particles get entrained in the prevailing aerodynamics and spread across the computational domain. Particles falling onto any surface get “trapped” onto the surface, while a “disappear” boundary condition is defined at outflows.

Table II lists down the different assumptions and simplifications that have been made while developing the CFD model.

**TABLE II.** The salient assumptions made in the model.

Sl. No.		Assumption
1	Geometry	The roof and back of the vehicle has been considered flat
2		The mouth print of the passengers has been considered as rectangular slits.
3	Model formulation	One way coupling of the velocity field on released particles has been considered
4		Effect of evaporation has not been considered
5		No breakage or coalescence of aerosol particles

### D. Grid independence test

**FIG. 2.**
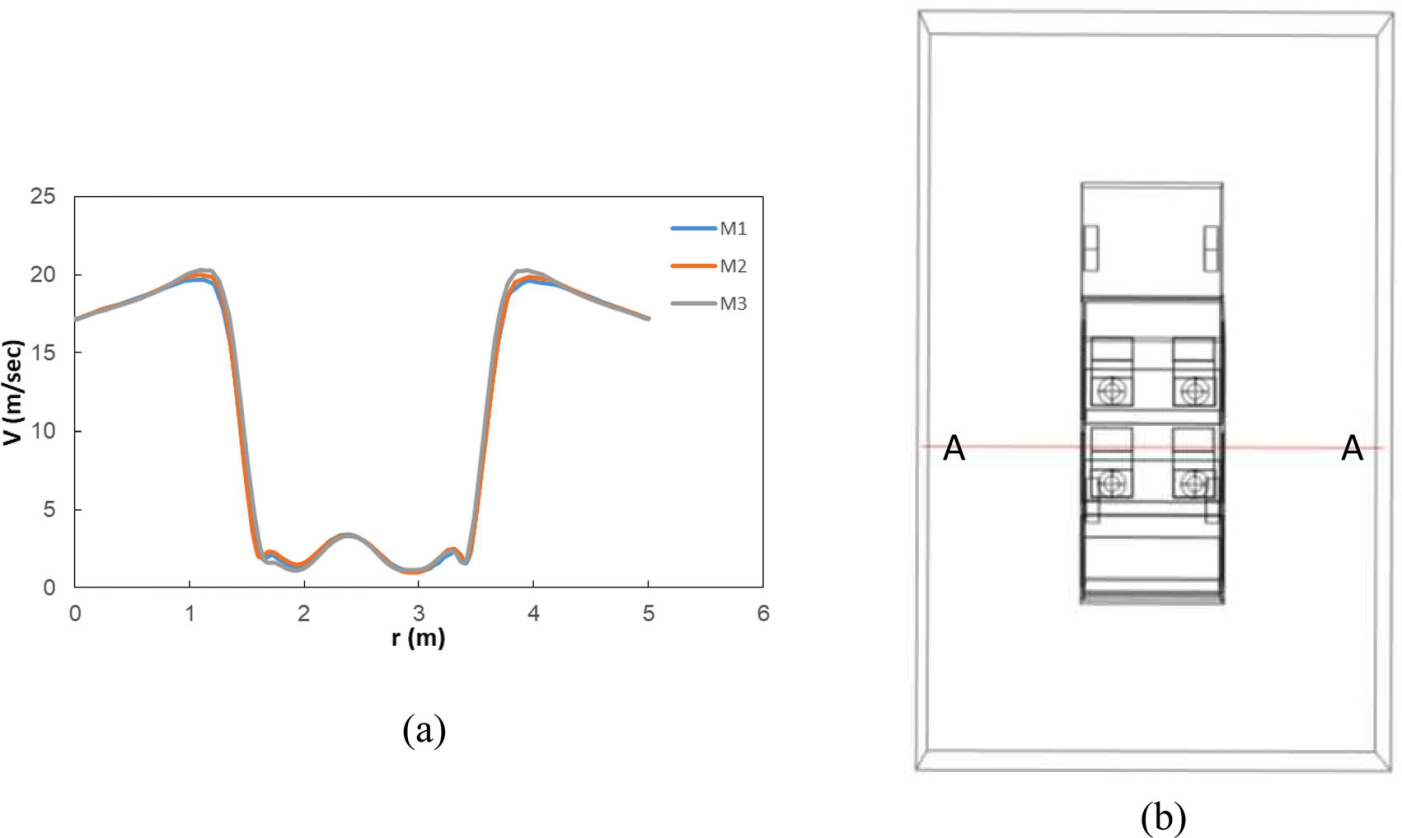
Results of grid independency test: (a) effect of grid density on the velocity profile along line AA and (b) location of line AA with respect to the computational domain.

The computational domain has been meshed using an unstructured tetrahedral mesh. The grid is non-uniform, and the regions near the mouth and nose of the seated persons have been refined. A mesh independency test has been carried out to quantify its effect on the flow field. Three mesh densities, namely, M1, M2, and M3 corresponding to 1.4 × 10^4^, 0.64 × 10^4^, and 0.5 × 10^4^ cells/m^3^ have been used. The result of the grid independence test is shown in Fig. 2. The parameter tracked is the variation of velocity along a line AA (elevation of 1.4 m from vehicle floor) as shown in Fig. 2(b). The results indicate that the velocity profile along AA is quite similar for all the three grid densities. Thus, a grid density of 6.4 × 10^3^ cells/m^3^ has been considered for further simulations.

## III. RESULTS AND DISCUSSION

### A. Different postulated scenarios

The objective of this work has been to study the state of dispersion of the micrometer-size aerosol particles generated by unhindered speaking of a person traveling in a moving SUV in the presence of other persons (co-passengers). The SUV is moving with its windows open and is exchanging air with the surrounding. Different scenarios pertaining to the vehicle speed, degree of opening of window, location of aerosol injection, and effect of cross flow have been modeled. Table III summarizes the different scenarios considered in simulations. It may be mentioned that for all scenarios, at a time only passenger speaks, while the others do not. For example, as per Table III, in scenarios 1–3, 8, and 9, only the driver (person A) speaks, while the others do not. In scenarios 4, 6, and 7, person C only speaks, while others do not. Finally, in scenario 5, only person D, speaks, while the others do not.

**TABLE III.** Different scenarios simulated.

Scenario No.	Purpose of simulation	Direction of wind	Windows open	Vehicle speed (km/h)	Person speaking
1	Effect of vehicle speed	From front	All	30	Driver (A)
2				60	
3				90	
4	Effect of location of person speaking	From front	All	60	Person sitting at left in the second row^a^ (C)
5					Person sitting at right in the second row^a^ (D)
6	Effect of window opening	From front	First and second row windows	60	Person sitting at left in the second row^a^ (C)
7			Only first row window		
8	Effect of cross flow	From left to right^a^	All	60	Driver (A)
9		From left to right^a^			

^a^ As seen from top.

As they follow the air currents, the particles can get trapped onto the internal surface of the vehicle or onto the bodies of the passengers, or they can simply move around and go out of the vehicle without getting deposited onto any surface. Indeed, particles falling onto the bodies of passengers and on the inner surface of the vehicle, which are frequently touched by the passenger, are important. However, in many cases, particles may move perilously close to the face of a passenger. In the present case, the mouth and nose of other passengers (except the one speaking) are defined as wall (zero velocity). However, in real life, people will be breathing in and out which means that in case a particle moves quite close to the face/mouth/nose of a passenger, it may be breathed in, which is a dangerous scenario. Thus, a risk parameter has been defined and assigned to each passenger (except the one speaking) in which a passenger is said to be at risk if any aerosol appears within an imaginary cubical enclosure of 0.5 m edge length, having its rear and bottom surface coincident with the rear and bottom surface of the spherical head of a seated human being (clarified in Fig. 6). This is a qualitative parameter which is set to “safe” when not a single micrometer-size particle enters this imaginary cube. In other words, as long as the aerosol path lines do not intersect with the boundaries of the cube, the corresponding passenger is safe. If any particle at any time enters this space, then the parameter is set to “at risk.” In addition, each scenario is quantified by the fraction of aerosols escaping the vehicle and the fraction trapped inside the vehicle (either onto the bodies of passengers or any other vehicle surface).

### B. Effect of particle size

**FIG. 3.**
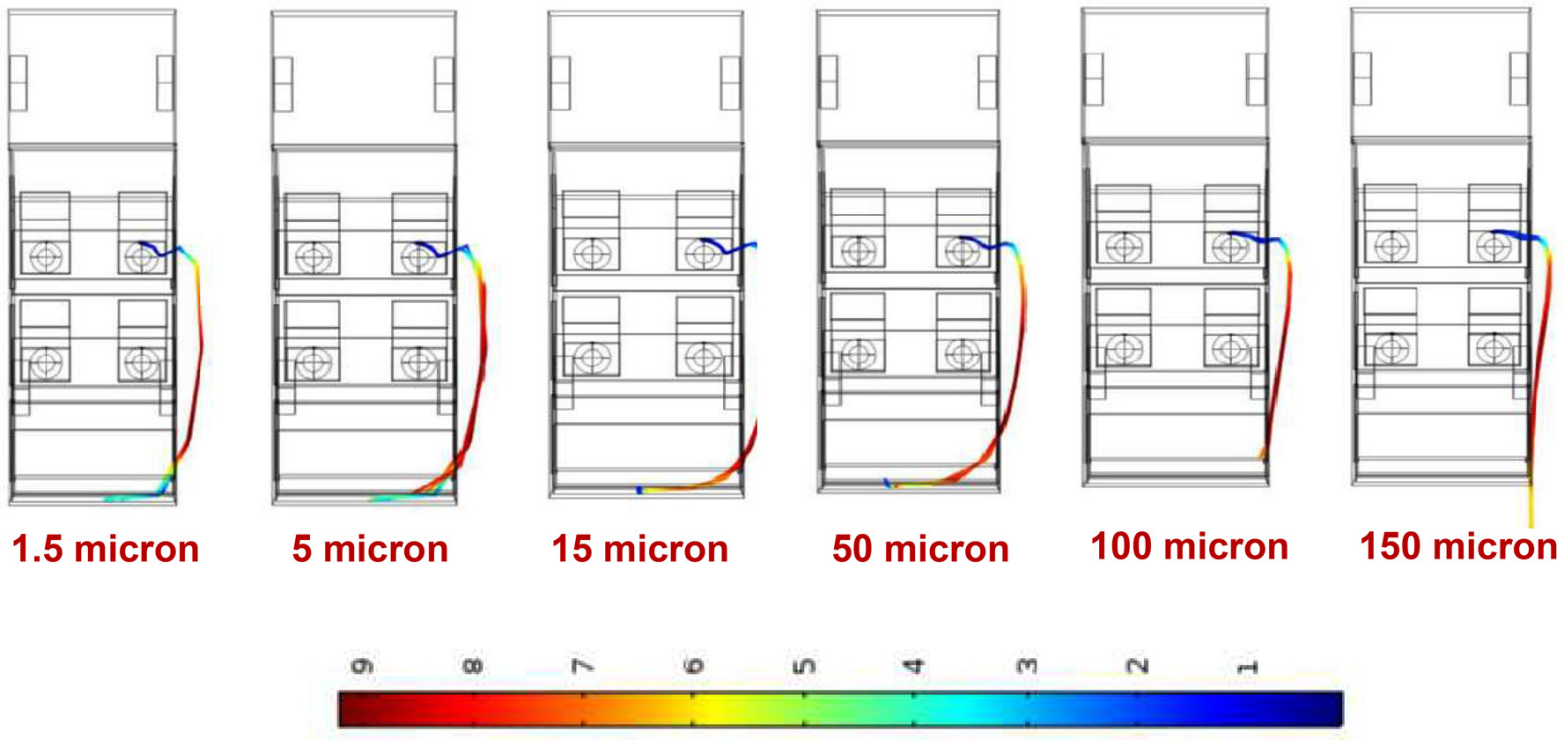
Effect of particle size on the state of dispersion. The color scale is that of particle velocity in m/s.

The particle size of the ejected aerosols will have a significant effect on the trajectories of particles as they are entrained in the prevailing aerodynamics. Typically, aerosols generated from drying out of respiratory droplets vary in the size range of 1–10 *μ*m (Narayanan and Yang, 2021). In this section, we study how particle size affects the state of dispersion. We have considered spherical particles in the size range of 1.5–150 *μ*m. Figure 3 shows the path lines of the particles as they are being ejected by person A (driver). Different path lines for different particle diameters are shown. The color shown is based on the local velocity of the particles. The condition simulated is that corresponding to scenario 2 in Table III (60 km/h speed, all windows open, air from front, and person A speaking).

The simulation reveals that the ejected aerosols will initially go out of the vehicle enclosure through the first window and then come back inside the vehicle through the third (rear) open window for particles up to 100 *μ*m size. However, beyond 100 *μ*m, the particles become too large to be dragged back into the vehicle. In fact for the case of 150 *μ*m sized particles, the inertia associated with the particles does not allow them to be dragged back inside the vehicle. The particles once outside continue their straight path and do not enter the vehicle. However, such large sizes are not typically associated with aerosols. It becomes evident from Fig. 3 that within the size range relevant to aerosols (below 10 *μ*m), there is no difference in terms of the path taken by them. Thus, the state of dispersion of aerosols can be modeled using any value of particle diameter in the range of 1 to 10 *μ*m. Another important inference that can be drawn is that in case some of the large diameter droplets (of the order of 100 *μ*m) do not get time to evaporate to residual aerosol of insoluble crystallites; they will go out of the vehicle and will not be any source of contamination. However, it is the smaller aerosol particles (<10 *μ*m) that actually re-enter the vehicle and are a cause of concern. Based on the results shown in this section, an aerosol size of 1.5 *μ*m has been chosen for further analysis of the different scenarios listed in Table III. This choice of particle size is also in line with the recent literature (Narayanan and Yang, 2021).

### C. Effect of vehicle speed (scenarios 1–3)

This section summarizes the key results from the simulations carried out to quantify the fate of the aerosols ejected due to person A speaking for the scenarios 1–3 detailed in Table III. In these scenarios, the vehicle speed is varied in the range of 30–90 km/h. This range is typical of any moving vehicle on highways. Four persons are seated, two each in first and second. The movement of the vehicle is mimicked by an air flow (at the same velocity) in opposite direction as discussed earlier.

**FIG. 4.**
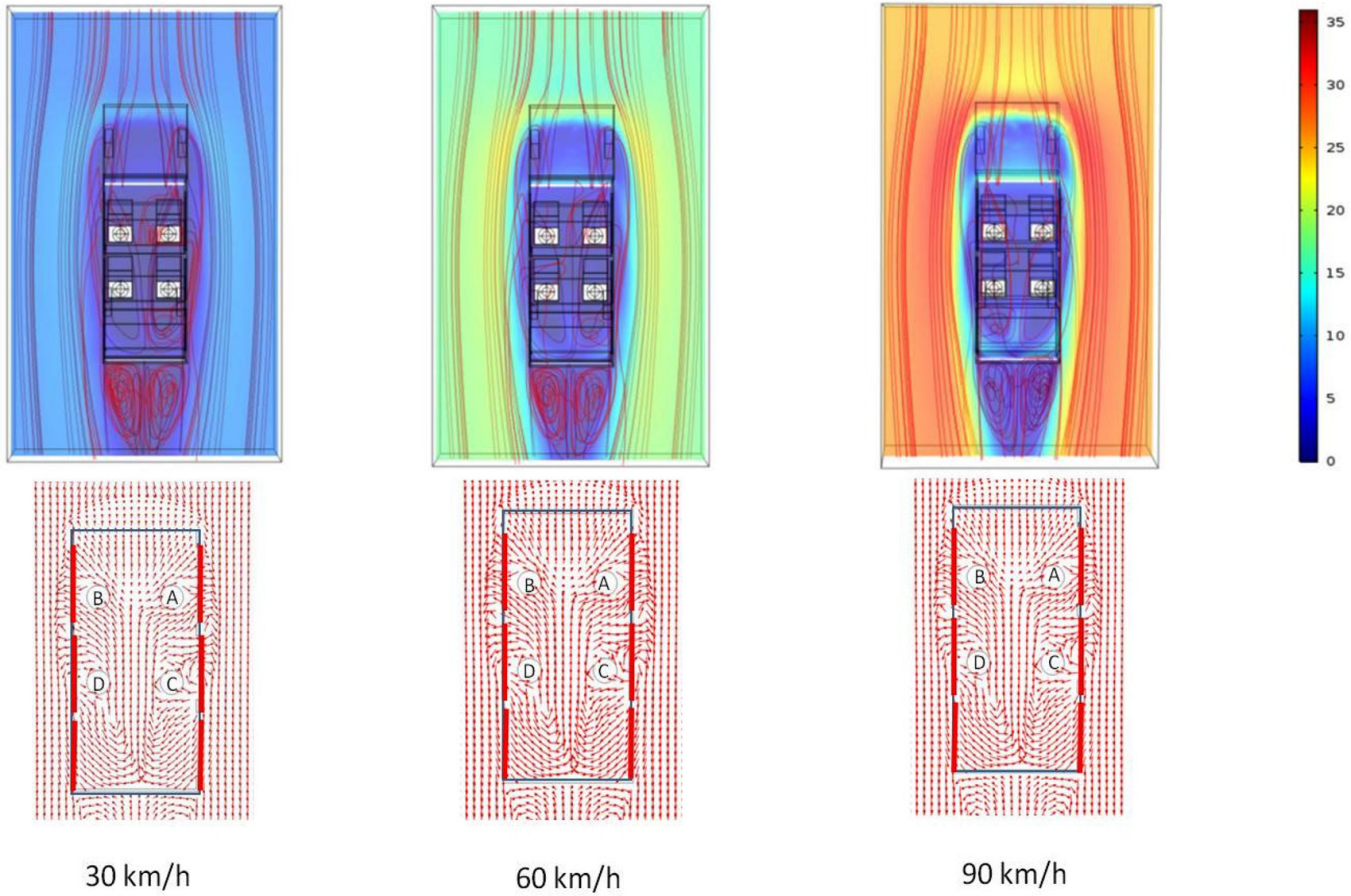
Spatial variation of velocity magnitude with streamlines (top panel) and normalized velocity vector plots (bottom panel) at a horizontal plane at the location of the mouth of the passengers at different vehicle speeds. The color scale is that of velocity in m/s (scenarios 1–3 from left to right).

**FIG. 5.**
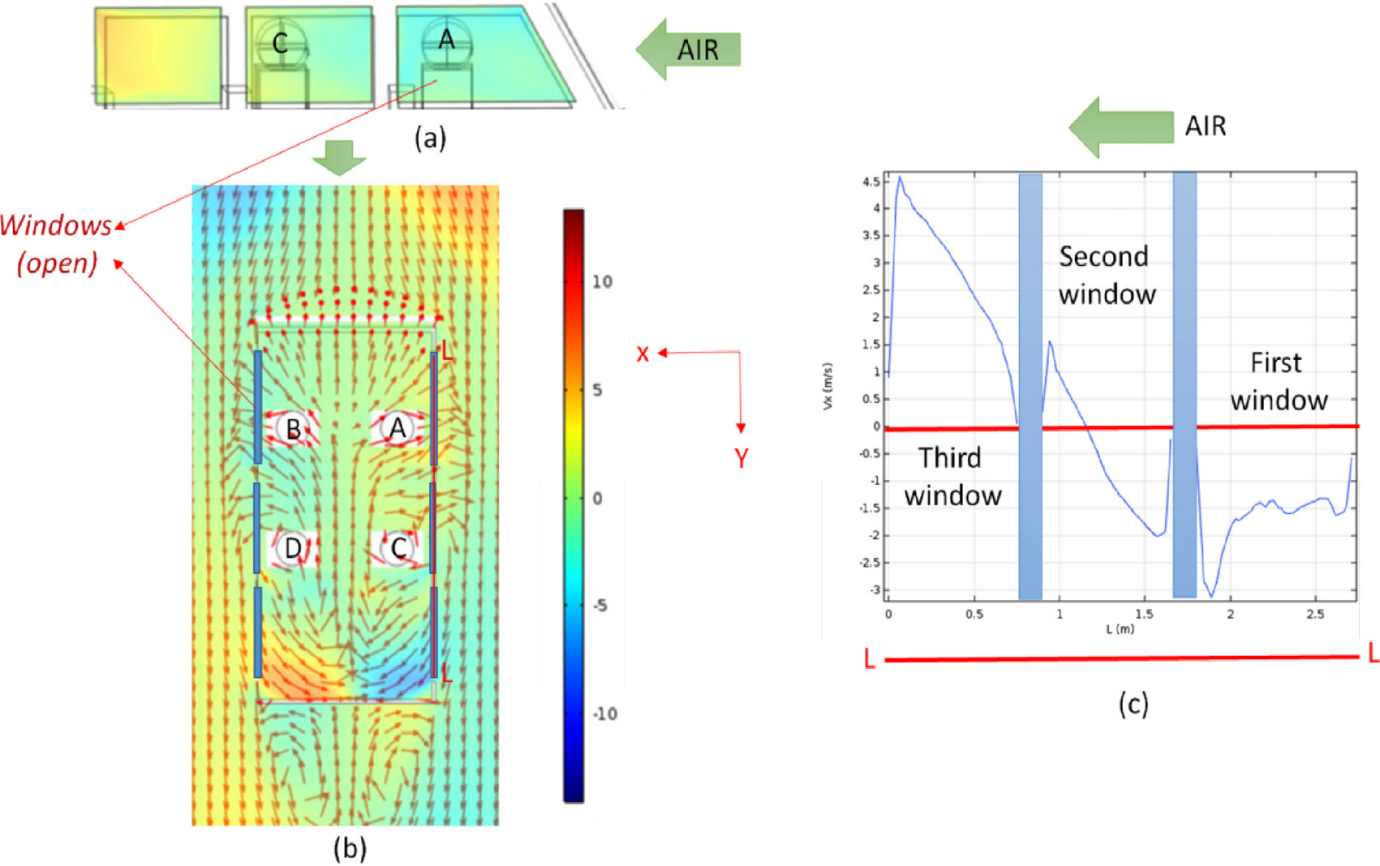
(a) Spatial variation of V_x_ in a vertical plane coinciding with the right side (in top view) windows, (b) spatial variation of V_x_ and velocity vectors in a horizontal plane at the height of the mouth of the passengers (color scale is that of X component of velocity in m/s), and (c) plot of V_x_ along a line LL running along the right hand side windows.

Figure 4 shows the velocity contour plot along with stream lines at a horizontal plane, which corresponds to the mouth of the passengers. As expected, the clear separation of boundary layers at the rear end of the vehicle is seen for all the three velocities. This leads to significant recirculations in the air just behind the vehicle (the wake region). Interestingly, counter rotating recirculations are also generated inside the vehicle. Such recirculation leads to significant churning inside the car interior, which will have a significant effect on the spread of the aerosols. The recirculations inside the vehicle are also clearly visible from the vector plots. The air currents tend to go out of the first window, while it comes back through the rear window. Such unconstrained recirculation is due to the opening of all the three rows of windows. This flow pattern is not only limited to one horizontal plane, but is present along longitudinal planes [Fig. 5(a)]. Figure 5(a) shows the contour of x component of the velocity vector along a vertical plane coincident with the right window(s) (as seen from the top). Values of V_x_ are negative in the region of the first row of window, signifying that air comes out of the vehicle through it. However, the values of V_x_ are positive in the region of the third row of window, signifying that the outside air rushes back into the vehicle through the rear window. This is also shown in Fig. 5(b) where the contour of V_x_ is shown on a horizontal plane coincident with the mouth of the passengers. Figure 5(c) quantitatively represents this phenomenon along a line (LL) which passes through the center of the right hand side window(s) as marked by red line in Fig. 5(b).

**FIG. 6.**
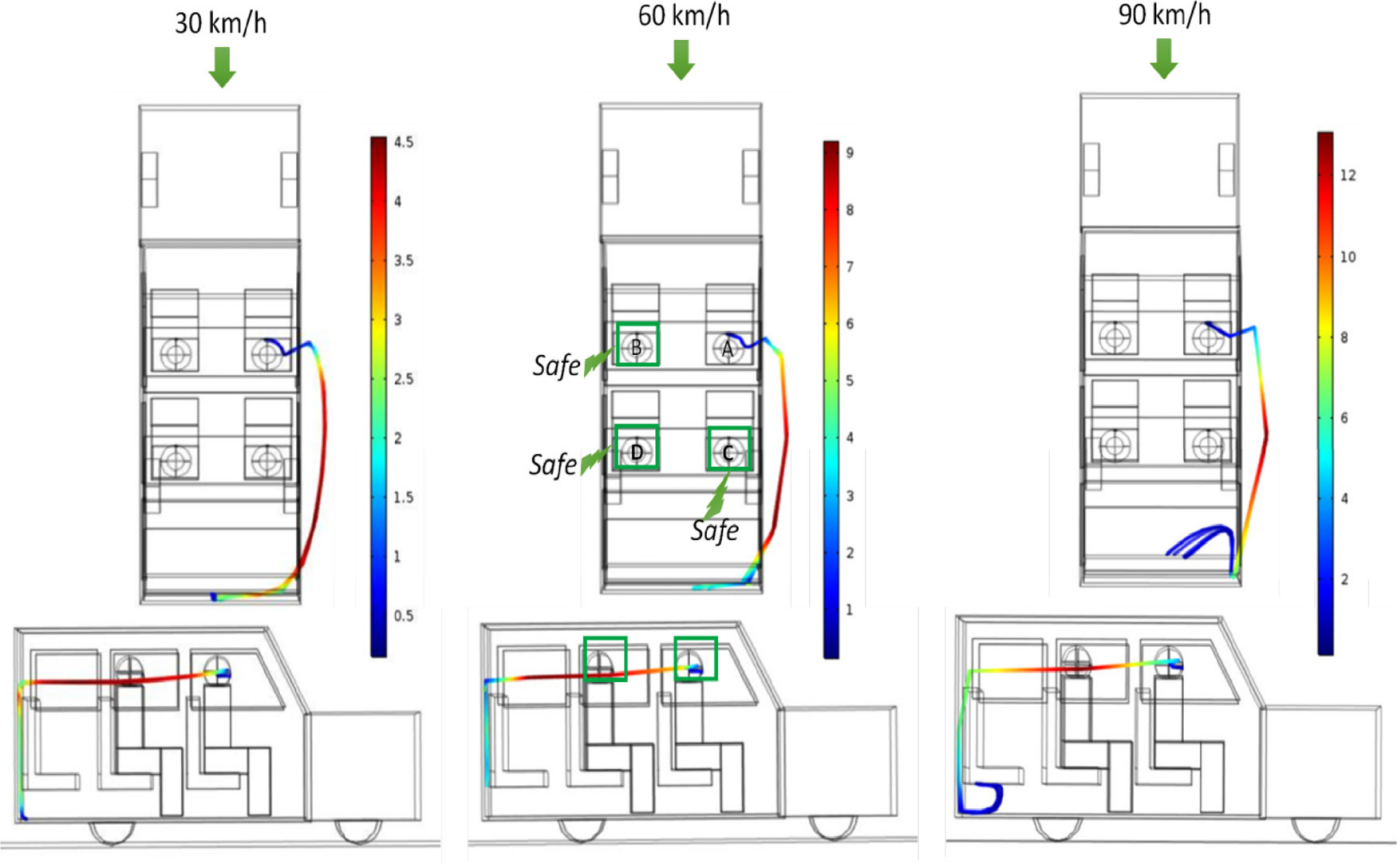
Particle trajectories of released aerosol as seen from top (top panel) and from sides (right hand side with respect to top view) (bottom panel): effect of different vehicle speed (scenarios 1–3 from left to right). The color scale is that of particle velocity in m/s.

Based on this prevailing flow pattern, the dispersion of the aerosols (1.5 *μ*m) is determined using the Lagrangian approach. Figure 6 shows how the aerosol particles circulate through the computational domain at three different vehicle speeds. Figure 6 shows the path lines of the particles from the top view as well as from the side view (right hand side as seen from the top). As can be seen, the path lines are rather similar for all the three velocities. In general, the aerosols move out of the first row of window and enter through the rear window (third row), and in doing so, they closely follow the air currents as shown earlier in Fig. 4. Some slight differences in path lines with respect to vehicle speed are apparent in Fig. 6 (right hand side view). At low velocities, particles enter through the rear window, strike the rear surface of the car, and are trapped there. However, at higher velocity (90 km/h), the particles moving with a higher velocity (as they are being dragged by the air stream moving at high velocity) tend to sweep through the back surface and are trapped at the bottom surface of the rear (third row) of seat. For the risk assessment, an imaginary cube of edge length 0.5 m is considered around the head of all other passengers except the one speaking. The location of the cube (imaginary) is so chosen in such a way that its bottom and rear surfaces coincide with the bottom and rear surfaces of the spherical head of a passenger. Thus, for scenarios 1–3, there will such cubes for person B, C, and D. Their relative position is shown in Fig. 6. As can be seen from the path lines, no aerosol particle enters the zone within these three cubes, and hence for scenarios 1–3 passengers B, C, and D are considered safe. This is shown for one of three cases (60 km/h) using green colored cubes which are also marked as safe. Moreover, a fraction of aerosol particles trapped onto the vehicle surface/bodies of passengers and that escaping the vehicle are also estimated. Table IV summarizes the results.

**TABLE IV.** Risk assessment for scenarios 1–3.

Scenario	Risk status				Fraction of aerosols trapped inside vehicle	Fraction of aerosols escaping vehicle
	A	B	C	D		
1 (30 km/h)	⋯	Safe	Safe	Safe	1	0
2 (60 km/h)	⋯	Safe	Safe	Safe	1	0
3 (90 km/h)	⋯	Safe	Safe	Safe	1	0

The simulation results indicate that even though all windows are open, there is a very high probability of aerosol particles being generated due to speaking, which circulate back inside the vehicle. Even though in our present study it does not put any of the passengers at risk, had there been any passenger in the last row, they would have been seriously affected. Another aspect is that vehicle speed does not have much of an effect in the way the aerosol particles tend to disperse. Thus, the spread of aerosols can be significant even at low vehicle speed.

### D. Effect of location of release of aerosol (scenarios 2, 4, 5)

This section summarizes the key results from the simulations carried out to quantify the fate of the aerosols ejected when a person speaks seated at different positions for scenarios 2, 4, and 5 described in Table III. In these scenarios, the vehicle speed is kept fixed at 60 km/h. The movement of the vehicle is mimicked by an air flow (at 60 km/h) in the opposite direction as discussed earlier.

**FIG. 7.**
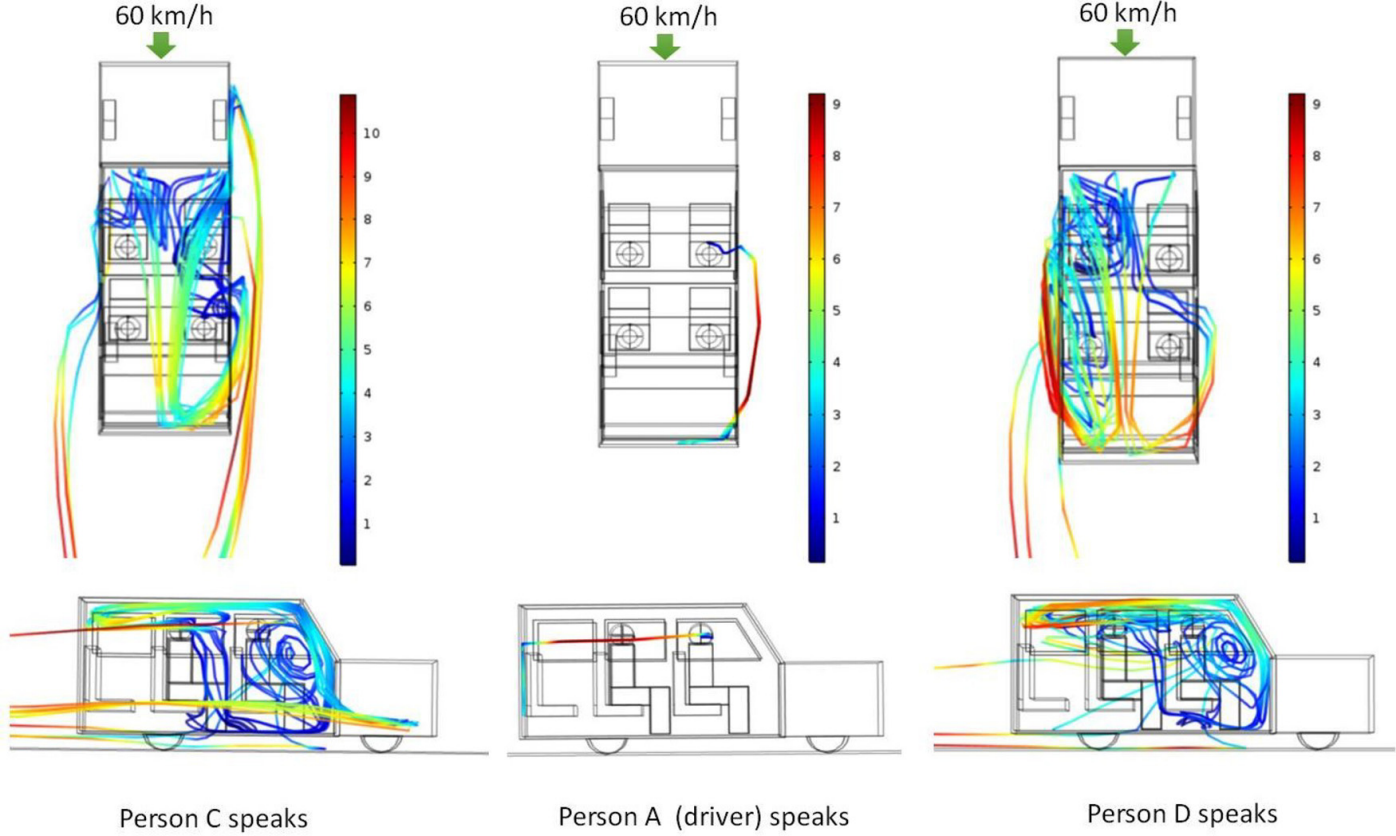
Particle trajectories of released aerosol as seen from top (top panel) and from sides (right hand side with respect to top view) (bottom panel): effect of location of aerosol injection (scenarios 4, 2, and 5 from left to right). The color scale is that of particle velocity in m/s.

Figure 7 shows the path lines of the ejected aerosols for three different cases—person C speaking, person A speaking, and person D speaking. It can be seen that while person A speaks, the aerosols move out of the vehicle through the first window and return back through the rear window. However, when person C and D (in second row) speaks, the ejected aerosol particles gets caught up in the recirculatory pattern that has been generated within the vehicle and circulate within the vehicle for a long time before eventually going out of the windows. This behavior can be understood from the velocity vector plot shown in Fig. 4 (60 km/h case). It can be seen that while the air predominantly moves out of the first row of window for the second row of window, some air moves out while some comes inside the vehicle. For the third row of window, the air comes back in. Thus, a local recirculation pattern exists between person C and D and the window (right or left respective, as seen from top). For scenario 4 (person C speaking), part of the aerosols get caught in this recirculation and start circulating within the vehicle, while the other portion goes out of the second (right) window and following the flow pattern comes back inside the vehicle through the third (right) window. However, unlike scenario 2, these particles do not hit the rear wall and come down. Instead they get entrained in the circulatory current (rear to front of the vehicle) that exists in the central region of the car (Fig. 4, 60 km/h case) and are transported toward the front of car. Finally, after significant churning in the front region of the car, they move outside through the front row of the windows (both left and right). During their transit, they intersect the imaginary cube around person A and B, thereby putting them at “risk.” Similarly, for scenario 5 (person D speaking), a portion of the aerosols move out of the vehicle from the second left window and come inside through the rear (left) window. Thereafter, similar to scenario 4, they get caught up in the central circulating current and are transported to the front of the car, wherein they undergo severe churning and eventually go out through the windows. As the particle move around, they intersect with the imaginary cubes around person A and B and put them at risk. Apart from the risk assessment, the fraction of particles trapped inside the vehicle and that escaping the vehicle are also estimated for scenarios 2, 4, and 5. Table V summarizes the results.

**TABLE V.** Risk assessment for scenarios 2, 4, and 5.

Scenario	Risk status				Fraction of aerosols trapped inside vehicle	Fraction of aerosols escaping vehicle
	A	B	C	D		
2 (person A speaking)	⋯	Safe	Safe	Safe	1	0
4 (person C speaking)	Risk	Risk	⋯	Safe	0.57	0.43
5 (person D speaking)	Risk	Risk	Safe	⋯	0.89	0.11

The simulation results show that chances of extensive circulation of aerosols within a moving vehicle with all its windows open are more when someone seated in the second row of seat speaks. Considering the results obtained so far (Tables IV and V), it may be said that for the case when all the windows are open and four persons are seated in a six seater SUV, chances of aerosol transmission from driver to passengers are much less as compared to that of passengers to the driver.

### E. Effect of number of windows opened (scenarios 4, 6, 7)

**FIG. 8.**
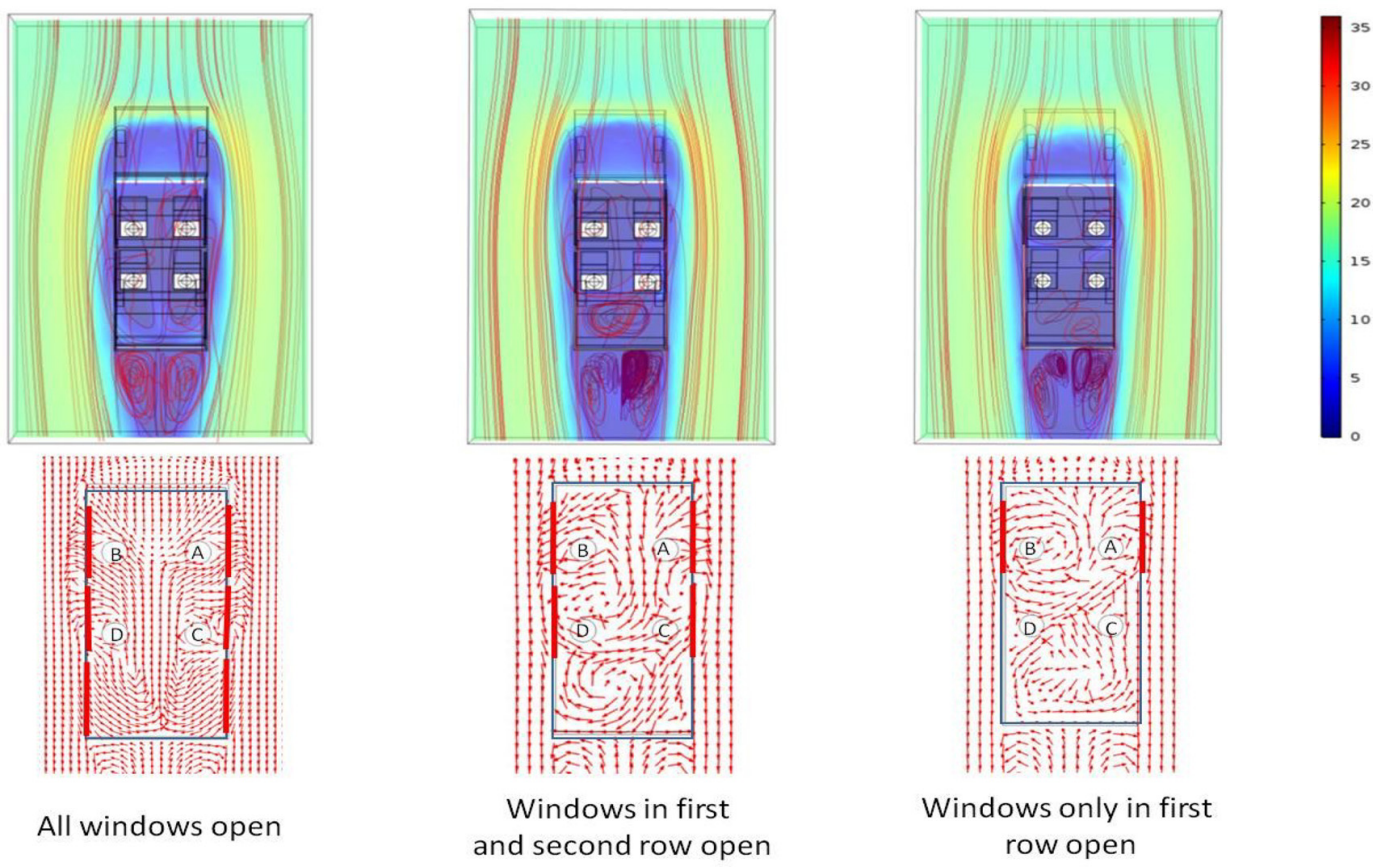
Spatial variation of velocity magnitude contour along with streamlines (top panel) and normalized velocity vector plots (bottom panel) in a horizontal plane at the height of the mouth of the passengers for different extents of window opening. The color scale is that of velocity in m/s (scenarios 4, 6, and 7 from left to right).

This section summarizes the key results from the simulations carried out to quantify the fate of the aerosols ejected during when person C speaks for scenarios 4, 6, and 7 described in Table III. In these scenarios, the vehicle speed is once again kept constant at 60 km/h. In these scenarios, we study what happens when the vehicle moves forward with less number of windows open. Earlier in Fig. 4 we have seen that a significant fraction of outside air enters the vehicle through the rear window. Thus, it is intuitive to ask what happens when the rear window is closed. Figure 8 shows the velocity contour plot along with stream lines at a horizontal plane which corresponds to the mouth of the passengers. As the vehicle speed is fixed at 60 km/h, the external aerodynamics (separation of boundary layer and formation of recirculations behind the vehicle) remains pretty much the same. However, there is significant difference in the flow patterns inside the vehicle. The well defined counter rotating recirculation spanning the entire length of the vehicle as seen for scenario 4 is replaced by a shorter counter rotating recirculation for scenario 6 (spanning a length roughly up to the second window), while for scenario 7 the span of the recirculation is the shortest (spanning the length of the first window only). However, it does not mean that the regions where the windows are closed are static. In scenarios 6 and 7, a rather large single circulation is observed in the region spanning the third row of seat. Some degree of mass exchange does occur amongst these recirculatory loops. This exchange is somewhat more pronounced for scenario 7. Such exchange means the aerosol particles can be transferred from one region to another region easily, thereby contaminating the entire car interior.

**FIG. 9.**
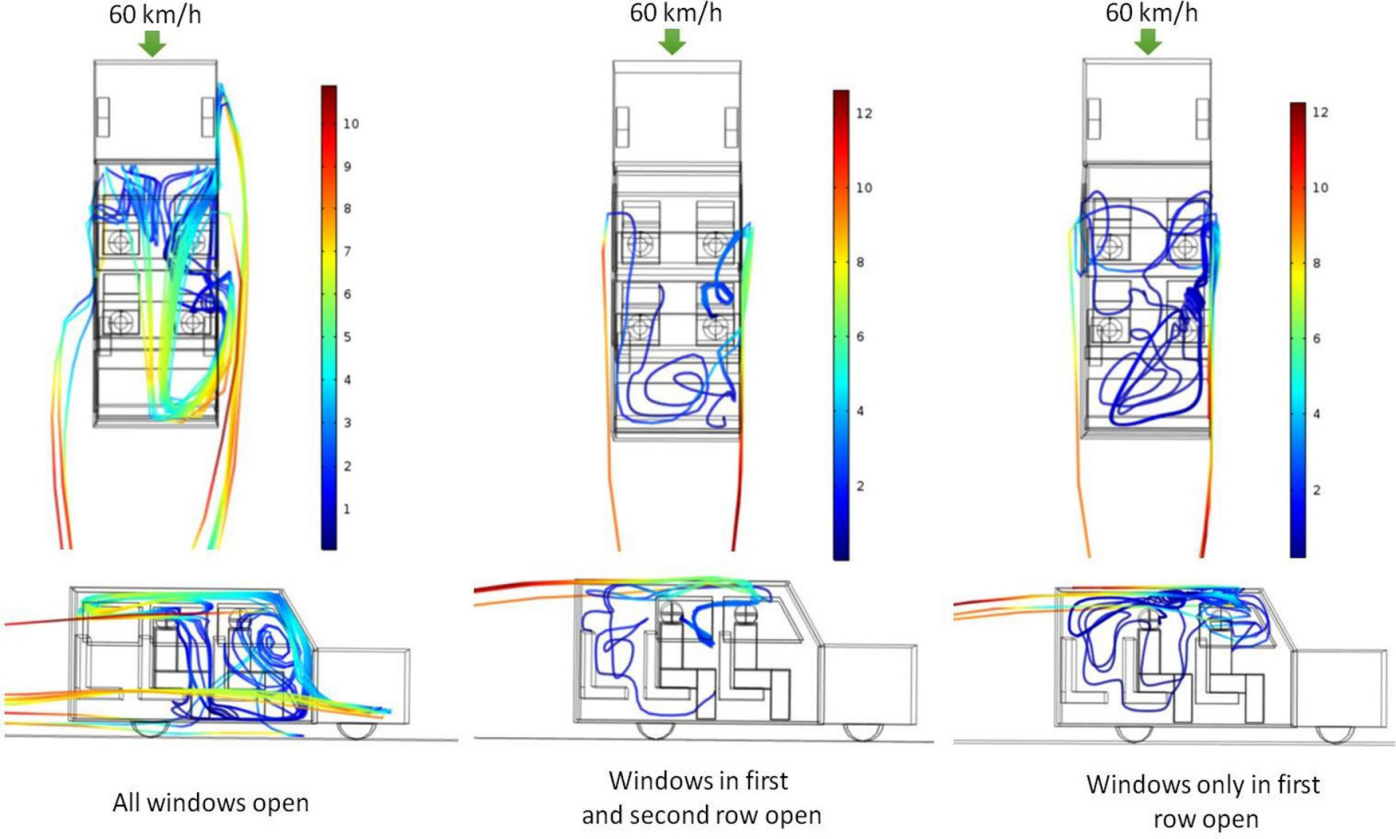
Particle trajectories of released aerosol as seen from top (top panel) and from sides (right hand side with respect to top view) (bottom panel): effect of different extents of window opening (scenarios 4, 6, and 7 from left to right).

Figure 9 shows the path lines of the ejected aerosols for three different cases—all windows open, windows of first and second row open, and windows of only first row open. In these cases, the person speaking is seated in the second row (person C). This case is chosen because the aerosol spread is much worse when a person in second row speaks. In scenario 4, part of the aerosols move out of the vehicle through the second window (right) and enter through the rear window (right). However, in scenario 6, the aerosol particles move forward and go out through the first window (right). This is because the velocity vectors for scenario 6 at the location person C are pointed forward toward the first (right) window (Fig. 8). A portion of the aerosols enter through the second (right) window, and once it enters, it gets caught up in the single recirculation loop generated in the rear portion of the vehicle (Fig. 8). However, the particles do not stick to any of the surfaces and are eventually transferred to the counter rotating recirculations (in the front end); hence, they are transmitted to the front section of the vehicle and finally go out through the first (left) window. For scenario 7, the ejected aerosols move toward the rear of the car as they get entrained in air currents which are moving backwards (Fig. 8, scenario 7, at location of person C). After significant churning in the rear portion of the car, the particles are transferred to the counter rotating recirculations in the front portion of the vehicle. Some particles are transferred to the left recirculation going out through the left window (first), while the other portion is transferred to the right recirculation going out through the right window (first). For both scenarios 6 and 7 during their transit across the entire vehicle cabin space, they intersect with the imaginary cubes around all persons (except person C), thereby putting all of them at risk. Apart from the risk assessment, the fraction of particles trapped inside the vehicle and that escaping the vehicle are also estimated for scenarios 4, 6, and 7. Table VI summarizes the results.

**TABLE VI.** Risk assessment for scenarios 4, 6, and 7.

Scenario	Risk status				Fraction of aerosols trapped inside vehicle	Fraction of aerosols escaping vehicle
	A	B	C	D		
4 (all windows open)	Risk	Risk	⋯	Safe	0.57	0.43
6 (windows in first two rows open)	Risk	Risk	⋯	Risk	0.11	0.89
7 (windows in first row only open)	Risk	Risk	⋯	Risk	0.86	0.14

The simulation results show that for the case of four persons traveling in a six seater SUV, it is advisable to open up all windows including the one at the rear. However, in any case if passengers seated at the second row speak unabated, there is significant chance of interpersonal transmission of aerosols. Based on the path lines for all cases up until now, it may also be stated that for all conditions, there is significant transport of aerosols toward the rear seat (third row). Thus, it may be advised that the rear seat is not occupied.

### F. Effect of cross winds (scenarios 2, 8, 9)

This section summarizes the key results from the simulations carried out to quantify the fate of the aerosols ejected when person A speaks for scenarios 2, 8, and 9 described in Table III. Up until now, all simulations carried out have been with the vehicle moving forward. However, another practical scenario can be that the vehicle is stationary (may be stuck in a traffic signal) with its windows open. During this period, there may be a gush of wind perpendicular to the car. In this section, this scenario has been considered, and the results for this case have been compared with the case when the car moves forward at 60 km/h (scenario 2). For consistency, wind velocity has been kept the same at 60 km/h.

**FIG. 10.**
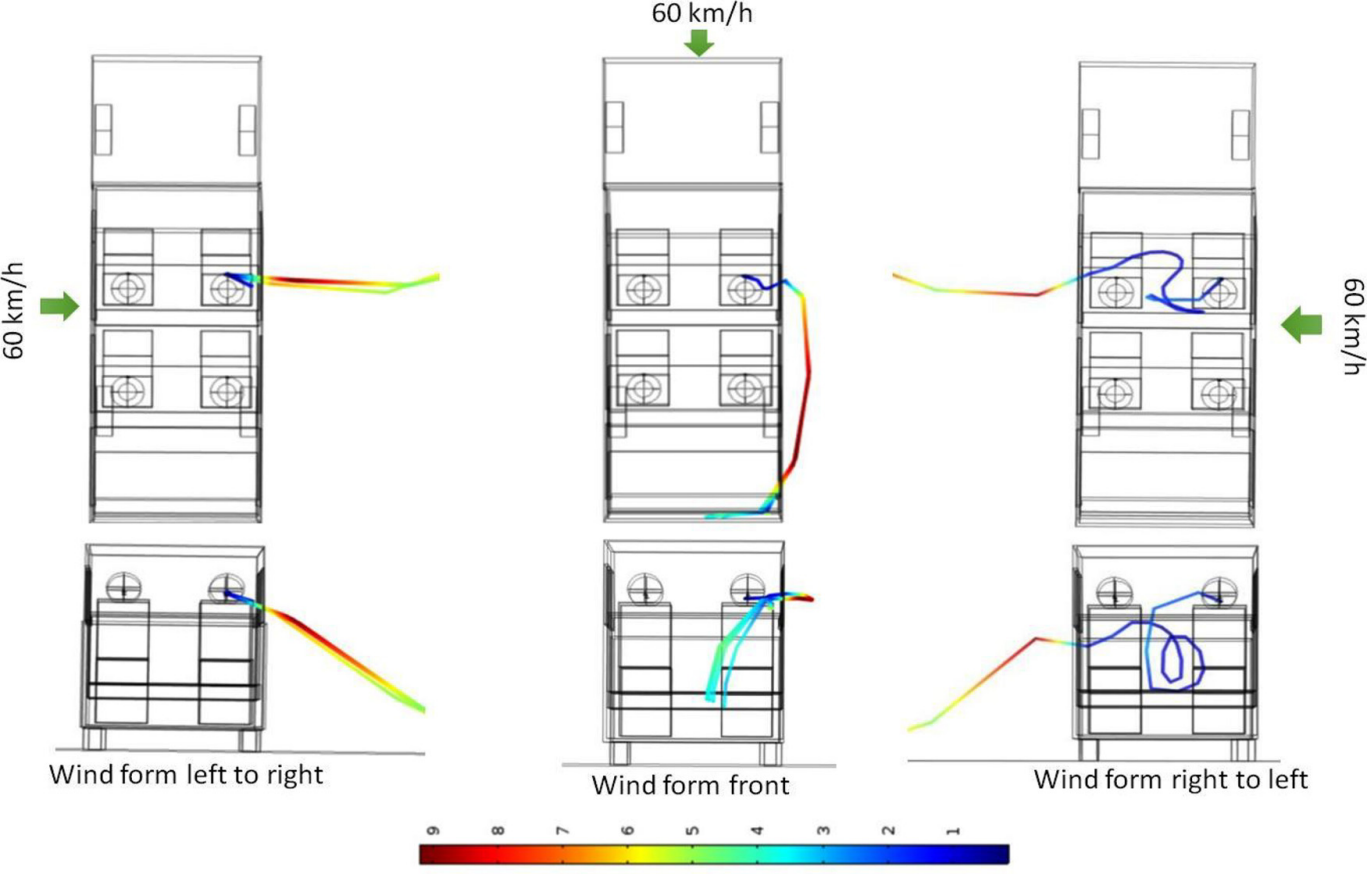
Particle trajectories of released aerosol as seen from top (top panel) and from rear of vehicle (bottom panel): effect of cross winds (scenarios 8 and 9 on left and right panel). Scenario 2 (middle panel) is included for comparison. Color scale is that of particle velocity in m/s.

**FIG. 11.**
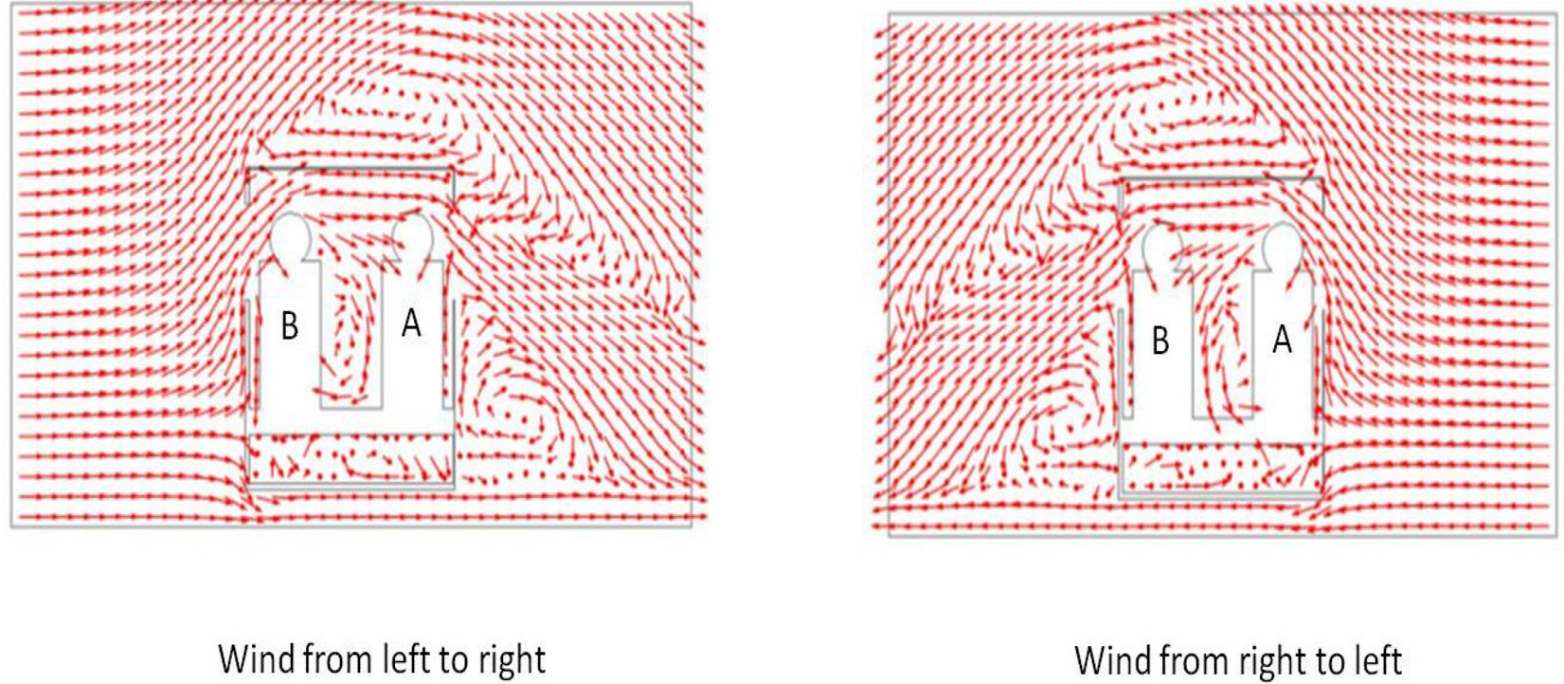
Normalized velocity vector plot at a vertical transverse plane coincident with person A and B as seen from the rear of vehicle for scenarios 8 (left panel) and 9 (right panel).

Figure 10 shows the path lines taken by the ejected aerosol particles for three different cases—cross wind from left to right (as seen from top), moving vehicle (at 60 km/h), and cross wind from right to left. For scenario 8 aerosol particles ejected by person A during speaking are flushed away through the first (right) window. Similarly for scenario 9, the aerosol particles are being carried away from right toward the left and go out through the left window. However, counter intuitively, on its way out though the first (left) window, they get caught in a recirculation and go out at an elevation at which they do not affect person B (the path lines do not intersect with the imaginary cube around person B). Thus, even though intuition suggests that person B may be at risk for scenario 9, simulation results indicate that this is not the case. Once outside, the aerosol particles tend to move downward and will eventually hit the ground/road. Figure 11 shows the normalized velocity vector plot in a vertical transverse plane passing through person A and B for scenarios 8 and 9. The transverse plane is seen from rear of the vehicle just like the case with Fig. 10 (bottom panel). The presence of recirculations on the leeward side of person A (scenario 9) is clearly seen, which is responsible for pulling down the ejected aerosol and ensuring that person B is safe. Moreover, just behind the vehicle, because of boundary layer separation, significant recirculations are seen which is responsible for a downward trajectory of the particle once they exit the vehicle.

Such path lines for scenarios 8 and 9 are thus quite different from scenario 2 where (for same wind velocity) particles goes out and then re-enters the vehicle. Thus, for the case of cross winds, all passengers are marked as safe. Apart from the risk assessment, the fraction of particles trapped inside the vehicle and that escaping the vehicle are also estimated for scenarios 2, 8, and 9. Table VII summarizes the results.

**TABLE VII.** Risk assessment for scenarios 2, 8, and 9.

Scenario	Risk status				Fraction of aerosols trapped inside vehicle	Fraction of aerosols escaping vehicle
	A	B	C	D		
2 (moving vehicle)	⋯	Safe	Safe	Safe	1	0
8 (cross wind left to right)	⋯	Safe	Safe	Safe	0	1
9 (cross wind right to left)	⋯	Safe	Safe	Safe	0	1

Thus, the simulation results show that passengers seated in a stationary vehicle (may be in a traffic signal) with open windows are safe in the presence of perpendicular cross winds irrespective of its direction.

**FIG. 12.**
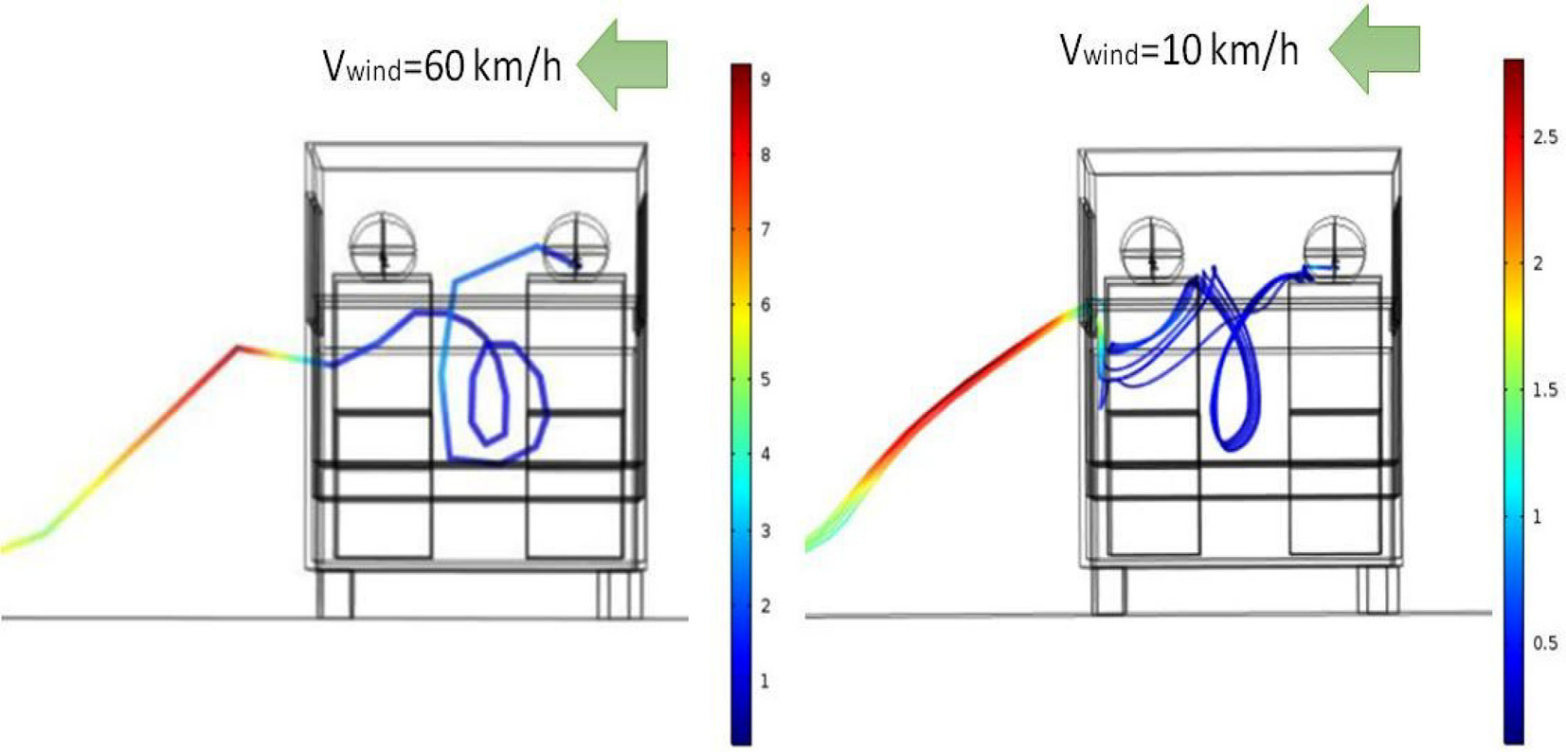
Particle trajectories of released aerosol as seen from rear of vehicle for two different cross wind (right to left) velocities. Color scale is that of particle velocity in m/s.

At this juncture, it may be argued that a cross wind velocity of 60 km/h is quite high, which is rather symbolic of stormy conditions in real life. Thus, it is intuitive to ask what happens when a light breeze blows across the vehicle as it is at rest. To answer this question, we carry out an additional simulation where the cross wind velocity is kept fixed at 10 km/h (symbolic of a light breeze) and study the dispersion of aerosols when person A (driver) speaks. Figure 12 compares the trajectories of the released aerosol particles at two different values of cross wind (from right to left)—60 and 10 km/h—as seen from rear of the vehicle. Figure 12 indicates that aerosol particles in both the cases follow similar trajectories in general even though some differences exist amongst them. The aerosols particles tend to be drawn toward person A to a greater extent at higher cross wind velocity (60 km/h) because of a stronger recirculation current on the leeward side of person A. Nonetheless even for the condition when a light breeze blows across the vehicle (from right to left, 10 km/h), person B (as well as C and D) is safe if person A (driver) speaks unabated.

## IV. CONCLUSIONS

A three dimensional (3D) Euler–Lagrangian model is used to simulate the dispersion of micrometer-size aerosols ejected while a person speaks inside a moving SUV. The windows of the SUV are open. As the vehicle moves forward with its windows open, there is an exchange of air within the vehicle with that outside. For the case where all windows are open, air inside the vehicle tends to go out through the first row of windows while the outside air rushes inside the vehicle through the rear (third row) window. This creates a well established countercurrent recirculatory flow inside the vehicle. Such prevailing aerodynamics will have a strong effect on the dispersion of aerosol particles that are generated while the passengers speak. Simulations revealed that state of dispersion of aerosol particles are not affected in the size range of 1–10 *μ*m, which is the typical size range of aerosols generated during speaking. The effect of different scenarios on dispersion of aerosols inside the vehicle, namely, vehicle speed, location of aerosol generation, number of windows opened, and effect of cross winds were studied. A risk parameter for each passenger was defined based on whether the aerosol path lines intersect an imaginary cube (0.5 m edge length) around the concerned person. Based on the results obtained in this work, the following conclusions can be made:

(a) Even at low vehicle speeds, small aerosol particles generated when the driver (person A) speaks first go out of the vehicle enclosure and come back inside even when all windows are open. Thus, the effect of vehicle speed did not have much of an effect on the general nature of dispersion of the aerosols.
(b) Chance of extensive dispersion of aerosol within the interior of the vehicle is much more if the person sitting in the second row of seats speaks in an unhindered manner. Thus, it can be said that chances of transmission of aerosols from driver to passengers is less than chances of transmission from passengers to driver. This is the scenario when all windows are open.
(c) When all windows in the car are open, it is advisable that the last row of seat be kept vacant as in all cases the results show significant dispersion of aerosols in the rear end of the vehicle.
(d) The dispersion of aerosols affects more passengers if a limited number of windows are opened. Thus, it is advisable to open all the three rows of windows while traveling.
(e) In case the car is stationary (as when at a traffic signal), the presence of cross winds does not have any detrimental effect on interpersonal transmission of aerosols.

The scenarios reported in this study are just a few of many more possible scenarios. For example, there could be a scenario in which the person speaking might bend his/her head up or down or toward another person in which case the aerosols will be ejected at an angle instead of being horizontal. Another scenario can be more than one person speaking and also facing each other. Yet another can be partial opening of the windows. Nevertheless, findings emanating from the simulations show how dispersion and spread of tiny aerosol particles is possible and significant even when all windows are kept open and there is a significant exchange of air within the vehicle.

## Data Availability

The data that support the findings of this study are available from the corresponding authors upon reasonable request.

## NOMENCLATURE

*C_d_*: Drag coefficient
*d_d_*: Particle diameter (m)
*g*: Acceleration due to gravity (m s^−2^)
*G_k_*: Turbulent kinetic energy generation rate (kg m^−1^ s^−3^)
*k*: Turbulent kinetic energy (m^2^ s^−2^)
*p*: Static pressure (Pa)
$\vec{u}$: Velocity vector of air (m s^−1^)
${\vec{u}}_{d}$: Particle velocity (m s^−1^)

**Greek letters**
*ε*: Turbulent energy dissipation rate (m^2^ s^−3^)
*μ*: Viscosity of air (Pa s)
*μT*: Turbulent viscosity (Pa s)
*ρ*: Density of air (kg m^−3^)
*ρ_d_*: Particle density (kg m^−3^)
$\overset{̿}{\tau }$: Total stress (Pa)
